# Longitudinal effects of green, blue, and gray spaces on early adolescent mental health in the United States

**DOI:** 10.1111/camh.12763

**Published:** 2025-03-24

**Authors:** Shannon Shaughnessy, Daniel Messinger, Spencer C. Evans

**Affiliations:** ^1^ Department of Psychology University of Miami Coral Gables FL USA

**Keywords:** Green space, blue space, gray space, adolescent psychopathology, latent growth curve model, Adolescent Brain Cognitive Development study

## Abstract

**Background:**

Physical environments are linked to adolescents' well‐being in various ways. Green and blue (natural) spaces may protect against psychopathology, while gray (urban) spaces may confer risk. The present study examines how exposure to green, blue, and gray spaces is associated with the growth of psychopathology in early adolescence.

**Method:**

We analyzed four waves of data (ages 9–13) from the Adolescent Brain Cognitive Development Study (*N* = 11,866, 47.8% female). At each wave, parents rated youths' mental health symptom severity in broad domains of total, externalizing, and internalizing problems. Latent growth curve models were estimated to model symptom trajectories. We examined the associations of residential proximity to green, blue, and gray spaces with symptoms at baseline and over time using geocoded and satellite data.

**Results:**

Green space was associated with lower levels of internalizing problems at baseline, while gray space was associated with higher levels of total and externalizing problems at baseline; however, all these effects diminished with time. Gray space was also associated with a slightly less positive slope for internalizing problems. There were no significant associations with blue space. Most results attenuated to nonsignificance once sociodemographic variables were accounted for.

**Conclusions:**

Green and gray space exposure may be positively and negatively associated with adolescents' psychosocial development, respectively. However, demographic variables such as gender and socioeconomic status may account for more change in early adolescent psychopathology than environmental variables. Regardless, greater attention to youths' green and gray space exposure could help promote mental health at a population level.

**Key practitioner message**

**What is known?**

- Proximity to natural (i.e., green and blue) spaces may be broadly beneficial for mental health, while proximity to urban (i.e., gray) spaces may be detrimental.
- No prior study has examined how green, blue, and urban spaces may be associated with the level and growth of psychopathology in early adolescence (ages 9–13).

**What is new?**

- In a representative US sample, higher exposure to green spaces, or less exposure to gray spaces, was associated with lower levels of emotional and behavioral problems; blue space had no significant associations.
- However, these effects were generally small, often diminished over time, and were mostly accounted for when we controlled for sociodemographic characteristics (child gender, race, and ethnicity; household income; and neighborhood disadvantage).

**What is significant for clinical practice?**

- Promoting youths' access to green spaces and reducing their exposure to gray space could help support mental health at the community level.
- It is important to consider the full context, as social, demographic, and economic characteristics may also be linked to youth mental health outcomes or explain the effect of physical spaces.

## Introduction

Adolescents are exposed to diverse environments. Some have ample access to *green spaces*, such as forests, trees, parks, and gardens (McCormick, 2017). Some live near *blue spaces*, or rivers, lakes, and oceans (Gascon et al., 2015). Some find themselves confined to predominantly *gray spaces* or built environments, including pavement. These environments influence adolescents' psychosocial well‐being, and these effects vary by the *type* of environment they are exposed to. Yet, to date, no study has examined how green, blue, and gray spaces may be associated with the growth and change in psychopathology symptoms throughout early adolescence (ages 9–13). The present study addresses this gap in the literature using data from the Adolescent Brain Cognitive Development (ABCD; Jernigan & Brown, 2018) study.

Increased green space exposure may have positive psychological impacts, as indicated by prior observational research. Proximity to green space in childhood is associated with reductions in hyperactivity, attention problems, and conduct problems (Bolanis et al., 2024; McCormick, 2017; Vanaken & Danckaerts, 2018). These effects may persist over time, as green space exposure in childhood is linked to a decreased risk of psychiatric disorders in adulthood, including personality, eating, depressive, and mood disorders, among others (Bezold et al., 2018; Engemann et al., 2019). In adolescence, green space exposure is associated with reduced stress, depressive symptoms, aggression, and psychological distress, as well as increased positive mood and mental well‐being (Bloemsma et al., 2022; Vanaken & Danckaerts, 2018; Zhang, Mavoa, Zhao, Raphael, & Smith, 2020). While some studies have found no effect of green space exposure on adolescent mental health, most evidence points to benefits (Mueller, Flouri, & Kokosi, 2019; Vanaken & Danckaerts, 2018), as these spaces may provide more opportunities for physical activity and social cohesion that improve mental health outcomes (Amoly et al., 2014). Of note, only a few of these prior studies have examined longitudinal change (de la Osa et al., 2024; Engemann et al., 2019; Putra, Astell‐Burt, Cliff, Vella, & Feng, 2021), and there is a dearth of research on early adolescence.

Research on the effects of blue spaces on adolescent mental health, particularly experimental studies, is limited but emerging (Bray, Reece, Sinnett, Martin, & Hayward, 2022; Díaz‐Martínez et al., 2023). Few adolescents, such as those in coastal communities, have access to bodies of water, while most have limited or no exposure. Preliminary evidence suggests that access to blue spaces improves mental health, well‐being, and physical activity in adulthood (Gascon, Zijlema, Vert, White, & Nieuwenhuijsen, 2017). In adolescence, blue space may be linked to lower emotional/behavioral problems and greater well‐being by providing more opportunities for stress‐reducing physical activity (Amoly et al., 2014; Huynh, Craig, Janssen, & Pickett, 2013). Other studies have found that blue space has no impact on mental health, but overall, there is a paucity of research in this area, particularly regarding potential longitudinal change (Bezold et al., 2018; Maes et al., 2021; Mavoa et al., 2019). Similarly to green space, there has been no study that examines whether blue space exposure is associated with the levels and growth of psychopathology throughout early adolescence.

Gray spaces, or urban, built environments, are associated with adolescent psychopathology symptoms. How these gray spaces are measured varies and may include geocoded variables or other metrics such as nighttime light exposure (Xu et al., 2022). Regardless of measurement, findings from previous observational studies indicate that adolescents in these environments are more likely to encounter violence, neighborhood disadvantage, crime, and noise pollution, which may be correlated with negative mental health outcomes, including potential increases in anxiety, depression, aggression, and posttraumatic stress (Mueller et al., 2019; Rudolph et al., 2019). Increased gray space exposure has also been linked to blunted biological stress reactivity and subsequent elevations in behavioral and emotional problems (Evans et al., 2020; Evans, Buil, Burk, Cillessen, & van Lier, 2018). However, characteristics of how gray spaces are built (e.g., streets, windows, and brick) may be just as important to adolescent mental health as indices of social disadvantage, and prior work has found associations between poorly built aspects of gray spaces and higher depression in adults (Galea, Ahern, Rudenstine, Wallace, & Vlahov, 2005). Of note, some studies suggest urbanicity is not detrimental to mental health and may even benefit social cognition (Breslau, Marshall, Pincus, & Brown, 2014; Xu et al., 2022). Yet overall, evidence suggests that increased exposure to urban environments may worsen adolescent mental health, although clarification on longitudinal effects during early adolescence is needed.

The present study investigated the associations of green, blue, and gray spaces with trajectories of psychopathology (internalizing, externalizing, and total problems) across early adolescence. We hypothesized that green and blue space exposures would be associated with more favorable mental health outcomes; conversely, gray space exposure would be associated with less favorable outcomes. We define *more favorable outcomes* as lower levels of symptoms at baseline and/or a steeper decline (or shallower increase) in symptoms over ages 9–13. We define *less favorable outcomes* as higher levels of symptoms at baseline and/or a steeper increase (or shallower decline) in symptoms over ages 9–13. Additional analyses were conducted to examine the specificity, robustness, and uniqueness of these associations, as well as to clarify the results for narrowband symptom domains (anxiety, depression, somatization, attention‐deficit/hyperactivity disorder [ADHD], oppositionality, and conduct problems).

## Method

### Participants

Analyses used data from ABCD, one of the largest and most representative longitudinal studies of adolescent development in the United States (Garavan et al., 2018; Jernigan & Brown, 2018). Recruitment was designed to be representative of the broader United States population. Advertisements were distributed to schools and local organizations via 21 research sites nationwide. To participate, children had to be between 9 and 10 years old at baseline, proficient in English, with no history of severe medical/neurological issues or legal blindness. In total, 11,880 children completed the baseline (Year 0) assessment. Of these, 11,866 children (99.88%; *M* age = 9.58, *SD* = 0.51; 47.8% female) were included in the present analyses, as they had at least one occasion of Child Behavior Checklist (CBCL; Achenbach & Rescorla, 2001) data and no more than one missing item on a subscale of interest. See Table 1 for detailed demographic characteristics. Rates of data availability for Years 0, 1, 2, and 3 were 100.00%, 94.44%, 68.10%, and 64.53%, respectively (averaging 18.23% missing data across waves; see Table 2 for details). The institutional review boards of each study site approved all study procedures.

**Table 1:** Child demographic characteristics for baseline sample (*N* = 11,866)

Variables	*N*	%
Biological sex		
Male	6187	52.14
Female	5676	47.83
Intersex – Male	3	0.03
Gender identity		
Male	6181	52.09
Female	5663	47.72
Transgender/Gender Queer	12	0.10
Refuse to answer/Unknown	10	0.08
Race		
White	7509	63.28
Black/African American	1867	15.73
American Indian/Native American	62	0.52
Chinese	70	0.59
Filipino	41	0.35
Asian Indian	52	0.44
Other race/Multiple races	2187	18.43
Refuse to answer/Unknown	78	0.66
Ethnicity		
Hispanic/Latino/a	2410	20.31
Not Hispanic/Latino/a	9303	78.40
Refuse to answer/Unknown	153	1.29
Household income		
Less than $5000	417	3.51
$5000–$15,999	694	5.85
$16,000–$34,999	1177	9.92
$35,000–$49,999	934	7.87
$50,000–$74,999	1498	12.62
$75,000–$99,999	1570	13.23
$100,000–$199,999	3310	27.89
$200,000 and greater	1250	10.53
Refuse to answer/Unknown	1016	8.56

The Transgender/Gender Queer category includes participants who identify as transgender female, transgender male, gender queer, and “other.”

**Table 2:** Data availability of longitudinal sample

Wave	Valid *N*	Dates of data collection	% of Baseline sample
Year 0 (ages 9–10)	11,866	2016–2018	100.00
Year 1 (ages 10–11)	11,203	2017–2019	94.44
Year 2 (ages 11–12)	8081	2018–2020	68.10
Year 3 (ages 12–13)	7657	2019–2021	64.53

Dates of data collection were obtained from https://abcdstudy.org/scientists/protocols/.

### Procedure

Baseline visits occurred in 2016–2018 (Garavan et al., 2018). After completing consent and assent forms with trained research assistants, caregivers completed questionnaires about demographics and psychopathology. Caregivers also provided the child's primary home address. In cases where the child spent less than 80% of their time at this primary address, up to two additional addresses could be provided (Goldblatt et al., 2024). Subsequent visits were structured similarly. Hybrid and remote testing options were made available in Years 2–3, after the onset of the COVID‐19 pandemic.

### Measures

#### Psychopathology symptoms

Psychopathology was measured using the CBCL, a parent‐report measure for children ages 6–18. The CBCL is one of the most common broadband measures of psychopathology and functioning and has demonstrated strong internal consistency, test–retest reliability, and convergent and discriminant validity with other informants and indicators of psychopathology (Achenbach & Rescorla, 2001). It was collected from the adolescents' caregivers at each time point. The CBCL consists of eight narrowband syndrome scales (e.g., thought problems and withdrawn/depressed), which form broadband measurements of *internalizing problems* (including anxious/depressed, withdrawn/depressed, and somatic complaints), *externalizing problems* (including rule‐breaking and aggressive behavior), and *total problems* (internalizing and externalizing, plus social, thought, and attention problems). In addition, the CBCL provides six DSM‐oriented subscales that are consistent with DSM diagnostic categories (i.e., affective problems [hereafter referred to as depression], anxiety, somatic problems, ADHD, oppositionality, and conduct problems). Raw sum scores were used in analyses. We examined the internalizing, externalizing, and total problem scales in our primary analyses and the six DSM‐oriented subscales in Appendix S1. Reliability across subscales and waves ranged from acceptable to excellent (*α*'s = 0.63–0.95; see Appendix S1 for exact values).

#### Urban‐satellite variables

To measure green, blue, and gray spaces, Urban‐Satellite variables were used (Goldblatt et al., 2024). These variables are derived from multiple sources of satellite and geocoded data that are based on participants' home addresses at baseline. For more information on how Urban‐Satellite variables are derived, please see Goldblatt et al. (2024). Primary addresses were used for analysis, as these were available for the full sample, and only a small portion (14.07%) listed a second address where they spent significant time.

*Green space* was measured with the *Normalized Difference Vegetation Index (NDVI)*. Higher NDVI values indicate denser and greener vegetation. For ABCD, the NDVI is calculated as the percentage of each pixel with an NDVI over 0.2, which indicates the presence of vegetation (range 0–1). The NDVI is commonly used in literature examining the impacts of green space exposure on adolescent mental health (Engemann et al., 2019).

*Blue space* was examined using permanent inland water area and seasonal water area, both of which are derived from the Copernicus Global Land Service categories (Buchhorn et al., 2020). *Permanent inland water area* is measured as the percentage of pixels within a grid cell that belong to this classification and ranges from 0 to 1. In contrast, *seasonal water area* is represented as a percentage of seasonal water coverage within a pixel, ranging from 0 to 1. For both variables, higher percentages indicate higher exposure to blue spaces.

*Gray space* was measured using built‐up land use and nighttime light exposure. *Built‐up land use* is derived from the Copernicus Global Land Service category and is expressed as a percentage of pixels (0–1) within a grid cell that belongs to this classification, with higher values indicating greater exposure to built‐up land (Buchhorn et al., 2020). *Nighttime light exposure* is derived from the Earth Observation Group (EOG) and is represented as the sum of the annual nighttime light radiance value within a pixel (possible range = 1 to 1005; Goldblatt et al., 2024). Higher values indicate greater exposure to nighttime lights, a metric of urbanicity.

#### Covariates

Consistent with prior literature (Vanaken & Danckaerts, 2018), sensitivity analyses controlled for the following relevant baseline covariates from parent‐reported demographic data: child *gender* (1 = female, 0 = else), *race* (dummy codes for white and black/African American, with multi/other as the reference group), *ethnicity* (1 = Hispanic/Latino, 0 = else), and *household income* (10 bins). In addition, we controlled for *neighborhood disadvantage* using the Area Deprivation Index (ADI), which considers factors such as housing quality, employment, and income using a participant's census tract (Fan et al., 2021; Kind et al., 2014). See Appendix S1 for additional information on covariates and their correlation with other study variables.

### Data analysis

Analyses were conducted using Mplus Version 8.0 (Muthen & Muthen, 2017). Latent growth curve models (LGCMs) were constructed to explore changes in psychopathology across early adolescence. LGCMs are a flexible analytical approach that allows simultaneous examination of between‐ and within‐person processes (Preacher et al., 2008). Full information maximum likelihood (FIML) estimation was used to address missing data, consistent with best practices (Enders & Bandalos, 2001). In all models, the latent intercept (factor loadings = 1) was specified to represent symptom levels at baseline (age 9.5), while the latent linear slope (factor loadings = 0, 1, 2, 3) was specified to represent symptom growth over time (from 9.5 to 12.5). All LGCM results are interpreted against several significance thresholds, emphasizing .05 as the field's standard convention and .001 as robust to type I error risk, given multiple comparisons.

Analyses involved carrying out LGCMs in four main steps. First, unconditional growth models were estimated to understand the basic pattern of change in symptoms over time. Second, conditional models were run with green, blue, and gray space predictors examined separately to assess their impact on psychopathology trajectories (intercepts and slopes). Third, the conditional models were re‐estimated with all the covariates included as predictors of the latent intercepts and slopes, allowing us to evaluate how the covariates affected the conditional results. Finally, we added all significant environmental predictors into a combined conditional model to assess the unique effects of green, blue, and gray space on total, internalizing, and externalizing problems (with and without covariates). Parallel supplementary analyses were conducted to examine results for specific symptom domains: depression, anxiety, somatic problems, ADHD, oppositionality, and conduct problems.

## Results

### Descriptive statistics, correlations, and unconditional models

**Figure 1:**
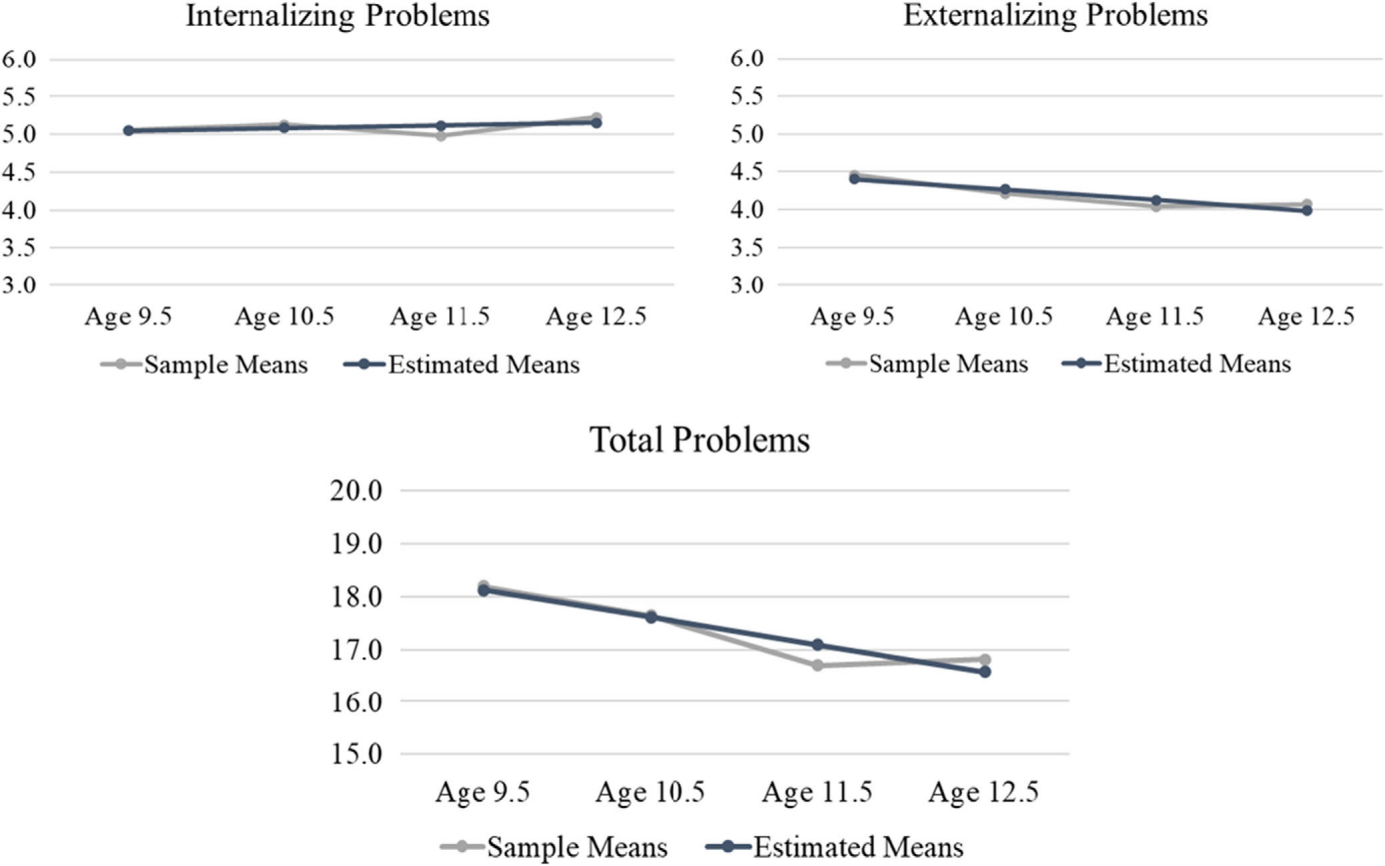
Observed means and model‐implied trajectories for unconditional growth models. See Table 3 for parameter estimates

See Appendix S1 and Tables S1 and S2 for descriptive statistics and correlations of all study variables. Unconditional LGCMs of internalizing, externalizing, and total problems are plotted in Figure 1, with model fit and parameter estimates reported in Table 3 (see Appendix S1 for corresponding results by DSM symptom domain). As shown, these linear models fit the data well, but patterns varied by symptom domain. Total (range across timepoints 0–161) and externalizing problems (range across timepoints 0–50) both started relatively low and decreased significantly over time (negative slopes, *p*s < .001). However, internalizing problems (range across timepoints 0–51) began low and showed a trend‐level increase over time (slope = 0.04, *p* = .056). In all three models, the variance terms for the latent intercepts and slopes were significant (*p*s < .001), indicating inter‐individual variability in symptom trajectories at baseline and over time. Finally, all six models yielded significant negative slope‐intercept correlations (*r*s = −.37 to −.22), indicating that youths' who had higher symptom severity at baseline tended to have more negative slopes of change over time (i.e., steeper decreases or less positive increases).

**Table 3:** Unconditional latent growth curve model results

Symptom trajectory	Estimate	*SE*	Est./*SE*	*p*‐Value	*χ* ^2^	CFI	RMSEA
Total problems					53.86***	0.99	0.03
Means							
Intercept	18.13	0.16	112.30	<.001			
Slope	−0.52	0.05	−10.24	<.001			
Variances							
Intercept	258.24	4.16	62.14	<.001			
Slope	8.48	0.48	17.71	<.001			
Slope‐intercept correlation	−0.33	0.02	−18.75	<.001			
Internalizing problems					43.91***	0.99	0.03
Means							
Intercept	5.05	0.05	102.45	<.001			
Slope	0.04	0.02	1.91	.056			
Variances							
Intercept	22.33	0.40	55.77	<.001			
Slope	1.05	0.06	16.71	<.001			
Slope‐intercept correlation	−0.22	0.02	−9.90	<.001			
Externalizing problems					46.77***	0.99	0.03
Means							
Intercept	4.41	0.05	83.95	<.001			
Slope	−0.14	0.02	−8.30	<.001			
Variances							
Intercept	26.77	0.44	60.61	<.001			
Slope	0.89	0.05	16.33	<.001			
Slope‐intercept correlation	−0.37	0.02	−20.95	<.001			

All estimates are unstandardized except for the slope‐intercept correlations; see Figure 1 for plots of observed means and model‐implied trajectories.

*** *p* < .001.

### Conditional models of environmental effects

All conditional models with green, blue, or gray space as predictors of psychopathology demonstrated good model fit (RMSEAs <0.05; CFIs >0.98; see Tables 4, 5, 6).

**Table 4:** Effects of green space (NDVI) on LGCM symptom trajectory growth terms

Models without covariates	Estimate	*SE*	Est./*SE*	*R* ^2^	*χ* ^2^	CFI	RMSEA
Total problems					48.74***	0.99	0.02
Intercept	−1.67***	0.46	−3.63	.001			
Slope	0.51***	0.14	3.55	.004			
Internalizing problems					44.40***	0.99	0.02
Intercept	−0.55***	0.14	−3.94	.002			
Slope	0.17***	0.05	3.29	.004			
Externalizing problems					41.37***	0.99	0.02
Intercept	−0.48***	0.15	−3.23	.001			
Slope	0.12*	0.05	2.54	.002			

Models with covariates	Estimate	*SE*	Est./*SE*	*R* ^2^	*χ* ^2^	CFI	RMSEA
Total problems					51.78***	0.99	0.01
Intercept	−0.67	0.51	−1.30	.06			
Slope	0.27	0.16	1.70	.05			
Internalizing problems					61.98***	0.99	0.02
Intercept	−0.30	0.16	−1.89	.03			
Slope	0.10	0.06	1.64	.05			
Externalizing problems					59.49***	0.99	0.02
Intercept	−0.19	0.17	−1.14	.07			
Slope	0.06	0.05	1.11	.03			

*p < .05, ***p < .001.

#### Green space

Greater proximity to green space predicted lower levels of total, internalizing, and externalizing problems at age 9.5. These effects were statistically significant but small, with reductions of about 1.67 points for total problems, 0.55 points for internalizing problems, and 0.48 points for externalizing problems for a one‐unit increase in NDVI (range: 0–1). Green space was positively associated with all three latent slopes (effects were 0.51, 0.17, and 0.12 for total, internalizing, and externalizing problems, respectively), indicating that the beneficial associations of green space at baseline diminished over time. Overall, green space accounted for only 0.1%–0.4% of the variance in these growth patterns (i.e., slope and intercept *R* ^2^s, Table 4).

All the above‐mentioned green space effects remained significant at the more stringent threshold of *p* < .001, except for the effect on externalizing problems' latent slope. However, when controlling for relevant covariates, none of these effects survived. Growth term *R* ^2^s suggest that these covariates accounted for a small but notable increase in variance accounted for, above and beyond green space, up to 3%–7% in total (see Table 4).

#### Blue space

Exposure to permanent or seasonal water was not associated with any symptoms at baseline or over time. The unconditional model *R* ^2^s were not appreciably different from zero; the conditional model *R* ^2^s again suggested that covariates alone accounted for 3%–7% of the variance in latent intercept and growth terms (see Table 5).

**Table 5:** Effects of blue space variables on LGCM symptom trajectory growth terms

Models without covariates	Estimate	*SE*	Est./*SE*	*R* ^2^	*χ* ^2^	CFI	RMSEA
Total problems					47.70***	0.99	0.02
Permanent water							
Intercept	2.96	4.19	0.71	0			
Slope	−0.77	1.26	−0.62	0			
Seasonal water					48.85***	0.99	0.02
Intercept	−2.06	8.00	−0.26	0			
Slope	1.28	2.49	0.52	0			
Internalizing problems							
Permanent water					41.63***	0.99	0.02
Intercept	0.61	1.28	0.47	0			
Slope	−0.27	0.46	−0.59	0			
Seasonal water					41.36***	0.99	0.02
Intercept	−0.69	2.45	−0.28	0			
Slope	−0.16	0.91	−0.17	0			
Externalizing problems							
Permanent water					40.58***	0.99	0.02
Intercept	0.46	1.36	0.34	0			
Slope	−0.27	0.42	−0.65	0			
Seasonal water					40.46***	0.99	0.02
Intercept	−0.89	2.59	−0.34	0			
Slope	0.44	0.83	0.53	0			

Models with covariates	Estimate	*SE*	Est./*SE*	*R* ^2^	*χ* ^2^	CFI	RMSEA
Total problems							
Permanent water					51.31***	0.99	0.01
Intercept	2.64	4.22	0.63	.06			
Slope	−0.98	1.28	−0.77	.05			
Seasonal water					51.60***	0.99	0.01
Intercept	−2.12	8.31	−0.26	.06			
Slope	1.45	2.65	0.55	.05			
Internalizing problems							
Permanent water					59.05***	0.99	0.02
Intercept	0.24	1.31	0.18	.03			
Slope	−0.30	0.47	−0.64	.05			
Seasonal water					58.90***	0.99	0.02
Intercept	−1.69	2.59	−0.65	.03			
Slope	−0.04	0.98	−0.05	.05			
Externalizing problems							
Permanent water					58.79***	0.99	0.02
Intercept	0.84	1.37	0.62	.07			
Slope	−0.33	0.43	−0.77	.03			
Seasonal water					58.42***	0.99	0.02
Intercept	−0.30	2.69	−0.11	.07			
Slope	0.61	0.89	0.68	.03			

***p < .001.

#### Gray space

Greater proximity to built‐up land use predicted higher levels of total problems at age 9.5 (Table 6). This effect was statistically significant but small, with increases of about 1.47 points for a one‐unit increase in built‐up land use (range 0–1), and this attenuated with age, eventually returning to average levels (the effect was −0.35). Greater proximity to built‐up land use also predicted higher levels of internalizing and externalizing problems at age 9.5. These effects were statistically significant but small, with increases of 0.41 points in internalizing problems and 0.48 points in externalizing problems for a one‐unit increase in built‐up land use. These effects persisted over time (i.e., nonsignificant effects on slopes).

**Table 6:** Effects of gray space variables on LGCM symptom trajectory growth terms

Models without covariates	Estimate	*SE*	Est./*SE*	*R* ^2^	*χ* ^2^	CFI	RMSEA
Total problems					50.93***	0.99	0.02
Built‐up land use							
Intercept	1.47**	0.49	2.99	.001			
Slope	−0.35*	0.15	−2.30	.002			
Nighttime lights					49.61***	0.99	0.02
Intercept	0.01***	0.002	4.65	.002			
Slope	−0.003***	0.001	−4.71	.01			
Internalizing problems							
Built‐up land use					41.89***	0.99	0.02
Intercept	0.41**	0.15	2.72	.001			
Slope	−0.09	0.06	−1.58	.001			
Nighttime lights					41.61***	0.99	0.02
Intercept	0.001*	0.001	2.34	.001			
Slope	−0.001***	0.000	−3.53	.004			
Externalizing problems							
Built‐up land use					41.98***	0.99	0.02
Intercept	0.48**	0.16	3.00	.001			
Slope	−0.08	0.05	−1.56	.001			
Nighttime lights					40.80***	0.99	0.02
Intercept	0.003***	0.001	5.15	.003			
Slope	−0.001***	0.000	−3.99	.01			

Models with covariates	Estimate	*SE*	Est./*SE*	*R* ^2^	*χ* ^2^	CFI	RMSEA
Total problems							
Built‐up land use					54.75***	0.99	0.01
Intercept	0.97	0.53	1.82	.06			
Slope	−0.11	0.17	−0.68	.05			
Nighttime lights					51.88***	0.99	0.01
Intercept	0.002	0.002	0.74	.06			
Slope	−0.001	0.001	−1.07	.05			
Internalizing problems							
Built‐up land use					58.66***	0.99	0.02
Intercept	0.40*	0.17	2.43	.03			
Slope	−0.01	0.06	−0.21	.05			
Nighttime lights					58.95***	0.99	0.02
Intercept	0.001	0.001	0.83	.03			
Slope	−0.0002	0.000	−0.63	.05			
Externalizing problems							
Built‐up land use					61.75***	0.99	0.02
Intercept	0.24	0.17	1.38	.07			
Slope	−0.01	0.06	−0.23	.03			
Nighttime lights					59.34***	0.99	0.02
Intercept	0.0003	0.001	0.43	.07			
Slope	−0.0003	0.000	−1.10	.03			

*p < .05, **p < .01, ***p < .001.

Greater proximity to nighttime light exposure predicted higher levels of total, internalizing, and externalizing problems at age 9.5 (.01‐, .001‐, and .003‐point increases, respectively, per a one‐unit increase in nighttime light exposure [range 1–1005]). Similar to built‐up land use, these effects were statistically significant but small and attenuated with age, as shown in their negative effects on latent slopes (−0.003, −0.001, and −0.001, respectively). Overall, gray space models accounted for 0.1%–1% of the variance in psychopathology growth terms.

Of all these gray space results, five effects were significant at *p <* .001: nighttime light exposure was positively associated with the intercept for total and externalizing problems and negatively associated with the slopes for all three variables. When controlling for relevant covariates, only the effect of built‐up land use on the intercept of internalizing problems remained significant. Again, covariate models accounted for 3%–7% of the variance in psychopathology growth terms.

### Combined models

Lastly, we estimated models that included all our green and gray space variables as predictors of psychopathology, as they showed significant associations in prior models. Blue space variables were omitted given that they showed no evidence of associations.

In the combined models, gray space predictors revealed significant effects only for nighttime light exposure; these seemed to overshadow any potential effects of built‐up land use (all nonsignificant; Table 7). Nighttime light exposure was significantly and uniquely associated with higher levels of total (0.01 increase) and externalizing (0.003 increase) problems at age 9.5, although these effects attenuated with time and eventually returned to average levels (decreases in 0.003 and 0.001 units over time, respectively). Interestingly, nighttime light exposure predicted a less positive slope in internalizing problems over time (decrease in 0.001 units over time) despite having no association at baseline.

**Table 7:** Results of combined model

Models without covariates	Estimate	*SE*	Est./*SE*	*R* ^2^	*χ* ^2^	CFI	RMSEA
Total problems					62.16***	0.99	0.02
Intercept				.003			
NDVI	−0.82	0.63	−1.30				
Nighttime lights	0.01**	0.003	3.09				
Built‐up land use	−0.25	0.70	−0.36				
Slope				.01			
NDVI	0.32	0.20	1.63				
Nighttime lights	−0.003***	0.001	−3.56				
Built‐up land use	0.28	0.21	1.31				
Internalizing problems					51.73***	0.99	0.02
Intercept				.002			
NDVI	−0.52**	0.19	−2.70				
Nighttime lights	0.0001	0.001	0.15				
Built‐up land use	0.03	0.21	0.13				
Slope				.01			
NDVI	0.16*	0.07	2.17				
Nighttime lights	−0.001*	0.000	−2.51				
Built‐up land use	0.12	0.08	1.59				
Externalizing problems					49.19***	0.99	0.02
Intercept				.003			
NDVI	−0.12	0.20	−0.57				
Nighttime lights	0.003***	0.001	3.87				
Built‐up land use	−0.07	0.23	−0.31				
Slope				.01			
NDVI	0.07	0.07	1.00				
Nighttime lights	−0.001***	0.000	−3.40				
Built‐up land use	0.10	0.07	1.36				

Models with covariates	Estimate	SE	Est./SE	*R* ^2^	*χ* ^2^	CFI	RMSEA
Total problems					59.48***	0.99	0.01
Intercept				.06			
NDVI	−0.24	0.67	−0.35				
Nighttime lights	−0.001	0.003	−0.40				
Built‐up land use	0.97	0.72	1.36				
Slope				.05			
NDVI	0.30	0.21	1.45				
Nighttime lights	−0.0004	0.001	−0.42				
Built‐up land use	0.12	0.22	0.55				
Internalizing problems					65.39***	0.99	0.01
Intercept				.03			
NDVI	−0.15	0.21	−0.72				
Nighttime lights	−0.001	0.001	−0.79				
Built‐up land use	0.40	0.22	1.80				
Slope				.05			
NDVI	0.14	0.08	1.79				
Nighttime lights	−0.0001	0.000	−0.11				
Built‐up land use	0.08	0.08	0.94				
Externalizing problems					66.25***	0.99	0.01
Intercept				.07			
NDVI	−0.11	0.22	−0.51				
Nighttime lights	−0.0005	0.001	−0.53				
Built‐up land use	0.23	0.23	1.00				
Slope				.03			
NDVI	0.07	0.07	0.94				
Nighttime lights	−0.0003	0.000	−0.88				
Built‐up land use	0.06	0.07	0.82				

*p < .05, **p < .01, ***p < .001.

Finally, green space (NDVI) was significantly and uniquely associated with lower levels of internalizing problems at age 9.5 (0.52 decrease), although these effects also attenuated over time and returned to mean levels (0.16 increase). However, green space was not uniquely associated with externalizing or total problems in these models, symptom domains that seem more directly impacted by nighttime light exposure, as described above.

When reexamining these results at a threshold of *p* < .001, only the effects of nighttime light exposure on externalizing problems and the slope of total problems remained significant. When controlling for relevant covariates, no results remained significant. The combined models accounted for 0.2%–1% of variance without covariates and 3%–7% of the variance with covariates. These patterns of *R* ^2^ estimates for the combined models are not appreciably different from the corresponding results for the separate models, again underscoring that the covariates play a bigger role than green, blue, or gray spaces in explaining psychopathology in early adolescence.

### Results by DSM symptom domain

As supplemental analyses, the above models were re‐estimated for specific DSM symptom areas as the outcome variables. Results showed that internalizing‐related subscales (anxiety, depression, somatization) and externalizing‐related subscales (ADHD, oppositionality, and conduct problems) largely followed patterns similar to those described above for CBCL internalizing and externalizing problem scales, respectively. At the same time, scale‐specific variations in these patterns emerged for all six DSM problem areas. Please see the Appendix S1 for detailed results on symptom‐level data as well as a summary of covariate effects.

## Discussion

The present study examined the impact of exposure to green, blue, and gray spaces on the trajectory of psychopathology during early adolescence. We hypothesized that increased proximity to green and blue spaces would be associated with *more favorable* mental health outcomes, while increased proximity to gray spaces would be associated with *less favorable* outcomes. These hypotheses were partially supported. Generally, results suggest favorable mental health effects associated with green space, none for blue space, and mixed effects for gray space. After accounting for covariates, most of these effects were attenuated to nonsignificance, highlighting the intricate interplay of context, environment, and mental health.

The results for green space exposure aligned with our hypotheses, showing mostly positive associations with mental health outcomes such as lower levels of total, internalizing, and externalizing problems at baseline. These effects, however, were modest and diminished over time, eventually returning to average levels. Interestingly, the beneficial nature of green space was most robust for *internalizing problems*, as these effects remained significant at both a significance value of .001 and within our combined model. Some prior work has found more robust associations between green space and internalizing problems than with externalizing problems (Madzia et al., 2019; Zhang et al., 2020). The explanations for these differential associations remain unclear. Green spaces may provide more opportunities for physical activity and social interactions that contribute to reductions in stress, anxiety, and depression; however, these pathways have not been investigated (Gascon et al., 2015; Putra et al., 2021). Future work should clarify potential differential associations between green space, externalizing problems, and internalizing problems and potential mechanisms underlying these effects.

All blue space effects were nonsignificant, suggesting minimal benefits of increased blue space proximity. Blue space is an emerging area of research with broad inconsistencies in measurement and conceptualization (Gascon et al., 2017). For instance, the variables we used for analyses focused on *inland* water bodies, omitting adolescents who may be exposed to blue spaces via the coast. There was also no clarification on the *type* of blue space, which may have weighed polluted water bodies as equal to safe freshwater bodies. Although outside of the scope of the present study, future work should aim to investigate the potential impact of blue space exposure by regional area (i.e., coastal or inland) and by type of space. Overall, more research on blue space proximity is needed to clarify any potential effects.

Exposure to gray spaces had mixed associations. Increased nighttime light exposure demonstrated a robust association with elevated total and externalizing problems at baseline, although these effects attenuated over time. Urbanicity's potential negative associations with adolescent mental health have been documented and are consistent with these results. Gray spaces may provide increased exposure to pollution, crime, and opportunities for sleep disruption, all of which are associated with negative health outcomes (Evans et al., 2018, 2020). However, we also found a robust association with gray space and the slope of internalizing problems, such that increased nighttime light exposure was associated with *a slightly less positive slope* (i.e., less steep increase) in internalizing problems over ages 9 to 13, contradictory to what we hypothesized. Urban environments may provide increased opportunities for assessment and treatment, which may, in turn, benefit internalizing symptoms. These environments may also provide more opportunities for social engagement and community participation for individuals with psychopathology, particularly when compared to rural environments, and this may help to improve symptoms over time (Townley, Brusilovskiy, & Salzer, 2017). Yet urbanicity may have only a marginally positive association with internalizing problems, as the significant effects of nighttime lights were small (slopes = −0.001), on an unconditional model slope that was only significant at a trend level (*p* = .056), and all effects attenuated to nonsignificance once we accounted for our covariates. Future work is needed to clarify the potential beneficial role of gray spaces on internalizing problems in early adolescence and elucidate mechanisms of these effects.

The results of our study underscore the importance of considering covariates in environmental research, as most of our findings were no longer significant after sensitivity analyses, and our covariates accounted for more variance in psychopathology than our environmental variables. Green, blue, and gray spaces cannot be examined in a vacuum, as they are often confounded by indicators of privilege, such as socioeconomic status. Potential associations between the physical environment and adolescent mental health may be crowded out by other drivers of psychopathology. Nonetheless, a small effect can have enormous implications when considered at the population level, and our results further contribute to clarifying the role of the environment in adolescent mental health (Carey, Ridler, Ford, & Stringaris, 2023).

The present study has some limitations. First, there may have been bias in the measurement of our variables, as psychopathology was only measured by parent report, and our blue space variables omitted exposure to coastal water, which may have impacted results. Our environmental and covariate variables were measured only at baseline, preventing us from examining how changes in these variables throughout early adolescence impacted mental health. For instance, a participant may have moved to an area with higher green space at age 12, and this may have had a positive effect on their mental health outcomes above and beyond their baseline green space exposure, but we are unable to parse this in the present study. Future studies should aim to include multiple informants of psychopathology, including adolescents themselves, and multiple measurements for green, blue, and gray spaces across multiple timepoints. Second, ABCD is an observational study, and we are unable to parse the exact effect of the environment on adolescent mental health. Future work should aim to examine similar questions within an experimental study, as these are largely absent from the broader literature. Third, although our study is representative of the larger United States population, it may not be representative of other countries, as the measured variables are context‐specific. Attrition in the ABCD study may also have impacted the generalizability of results (Ewing et al., 2022). Fourth, given our high number of analyses, there is a risk for type 1 errors (i.e., false positives). Results reported with the .001 threshold for significance should be regarded as the most reliable and robust to address these concerns. The present study also has several strengths. We had a large, well‐powered sample size, which allowed us to detect small, meaningful effects. In addition, although this may have been impacted by attrition, our results are largely generalizable to American adolescents as participants in the ABCD study are representative of the US population (Garavan et al., 2018). We also leveraged a strong analytic approach, as LGCM allowed us to examine growth over time, which would have been lost in standard longitudinal analyses.

## Conclusion

Adolescents interact with diverse environments every day. These environments may have powerful associations with their mental health, with largely *positive associations* for green spaces and largely *negative associations* for gray spaces. Yet future work is needed to delineate the exact direction of these effects and the potential confounding by demographic variables. Relatedly, future work should examine how these demographic variables (e.g., socioeconomic status) may be a driver of differential environmental exposures. Randomized control trials (RCTs) and quasi‐natural experimental studies would be particularly beneficial, as results from these could elucidate the therapeutic effects of engaging in these spaces. By developing these therapies and thoughtfully engineering our urban spaces, we stand to greatly improve the lives and mental health of the adolescents around us.

## Funding information

The present study was not supported by any external funding sources.

## Conflict of interest statement

The authors have no conflicts of interest to disclose.

## Ethics statement

All study procedures were approved by the respective ABCD study sites. Relevant consent and assent were obtained for all participants.

## Supporting information


**Appendix S1.** Supporting information.
**Figure S1.** Observed means and model‐implied trajectories for unconditional growth models of symptoms.
**Table S1.** Descriptive statistics of study variables.
**Table S2.** Correlations among study variables at baseline.
**Table S3.** Unconditional latent growth curve model results.
**Table S4.** Effects of green space (NDVI) on LGCM symptom trajectory growth terms.
**Table S5.** Effects of blue space variables on LGCM symptom trajectory growth terms.
**Table S6.** Effects of gray space variables on LGCM symptom trajectory growth terms.

## Data Availability

The present study used data obtained from the Adolescent Brain Cognitive Development (ABCD) study for all analyses, and relevant permissions were obtained for its use. For more information on obtaining access to ABCD data, please visit https://nda.nih.gov/abcd. Mplus code for analyses can be found at DOI 10.17605/OSF.IO/VFBXH.
