# Supplementary material for: Longitudinal effects of green, blue, and gray spaces on early adolescent mental health in the United States
Source: Child Adolesc Ment Health. 2025 Mar 24;30(2):119–30. doi: 10.1111/camh.12763 (PMC12079718; doi:10.1111/camh.12763)
Supplement: Supplementary file 1 — Appendix S1. Supporting information. Figure S1. Observed means and model‐implied trajectories for unconditional growth models of symptoms. Table S1. Descriptive statistics of study variables. Table S2. Correlations among study variables at baseline. Table S3. Unconditional latent growth curve model results. Table S4. Effects of green space (NDVI) on LGCM symptom trajectory growth terms. Table S5. Effects of blue space variables on LGCM symptom trajectory growth terms. Table S6. Effects of gray space variables on LGCM symptom trajectory growth terms. [file CAMH-30-119-s001.docx]

**Supporting Information**

**Descriptive Statistics and Correlations Over Time**

On average, parent-reported psychopathology was low (see Table S1). In terms of environmental variables, there was a low presence of permanent (*M* = 0.01) and seasonal water (*M* = 0.01). All psychopathology scales were positively correlated (*rs =* 0.22 to 0.89; see Table S2). Green space was negatively correlated with total, internalizing and externalizing problems as well as anxiety, depression, somatization, oppositionality, and conduct problems (not with ADHD). Grey space was positively correlated with total, internalizing, and externalizing problems as well as with depression, ADHD, oppositionality, and conduct problems (not with anxiety or somatization). Blue space was not correlated with any form of psychopathology symptoms. The green, blue, and grey space variables ranged from being entirely unrelated (NDVI with seasonal water) to large correlations among NDVI, land use, and light exposure (*r*s = -0.66 to -0.55).

**Results of Symptom-Level Models**

**Unconditional Latent Growth Curve Models.** All unconditional models demonstrated good overall fit (RMSEAs < .05; CFIs > 0.98; see Table S3). As shown in Figure S1, these model-implied trajectories corresponded closely to the observed means at each wave. These results indicated that the data can be represented as linear patterns of change over time. For five of the six symptom variables—anxiety, somatization, ADHD, oppositionality, and conduct problems—average trajectories started at low (but non-zero) levels of symptoms at baseline (intercepts = 1.08-2.60, *p*s<.001), and then decreased significantly over time (slopes = -0.10 to -0.02, *p*s<.001). The exception to this pattern was depression, which also started low (intercept = 1.26. *p*<.001), but then *increased* significantly over time (slope = 0.14, *p*<.001). In all six models, the variance terms for latent intercepts and slopes were significant (*p*s<.001), indicating inter-individual variability in symptom trajectories at baseline and over time. Finally, all six models yielded significant negative slope-intercept correlations (*r*s = -.38 to -.18, *p*s<.001). In other words, youths who started at higher levels of symptom severity at baseline tended to have more negative slopes of change over time (i.e., steeper decreases or less positive increases).

**Conditional Latent Growth Curve Models.** All conditional growth curve models with green space as a predictor demonstrated good model fit (RMSEAs < .05; CFIs > 0.98; see Table S4). Greater proximity to green space (as measured by the NDVI) predicted lower levels of anxiety, depression, somatization, oppositionality, and conduct problems at age 9.5. These effects were statistically significant but small, with reductions of about 0.10-0.19 points in baseline CBCL symptom scores (scale ranges: 0 to 10+) for a one-unit increase in NDVI (range: 0 to 1). Over time, the positive effects on depression and conduct problems persisted (i.e., nonsignificant effects on slopes). For anxiety, somatization, and oppositionality, the benefits at baseline diminished over time, with a slower decline in symptoms over time (anxiety= 0.06 points/year; somatization = 0.03 points/year; oppositionality= 0.06 points/year), returning to average levels after a few years. Interestingly, green space was positively associated with the latent slope for ADHD but not its intercept, indicating that green space did not have concurrent effects on ADHD at age 9.5 but was linked to a less steep decline in symptoms over time (0.06 points/year). When reexamining these results at a threshold of *p* < .001, only the significant effects of green space on baseline depression and the growth of oppositionality remain significant. In addition, when controlling for relevant covariates, only the significant effects of green space on baseline levels of somatization (intercept = -0.09, SE = 0.04, *p* < .05) and on the growth of oppositionality (slope = 0.04, SE = 0.02, *p* < .05) remained, with neither of these significant at a threshold of *p* < .001.

All conditional growth models with blue space as a predictor demonstrated good model fit (RMSEAs < .05; CFIs > 0.98; see Table S5). Exposure to permanent or seasonal water did not predict any symptoms at baseline or over time.

All conditional growth models with grey space as a predictor demonstrated good model fit (RMSEAs < .05; CFIs > 0.98; see Table S6). Increased exposure to built-up land use was associated with higher levels of depression (intercept = 0.14) and conduct problems (intercept = 0.18) at age 9.5, with these effects persisting over time (no effects on slopes). Increased proximity to built-up land use was linked to a faster decline in ADHD symptoms over time (slope = -0.06). Increased nighttime light exposure was positively associated with higher intercepts for depression, somatization, ADHD, oppositionality, and conduct symptoms at age 9.5 (effects = 0.0004-0.002 higher symptom scores per one-unit increase in nighttime light, range 1-1005), but these effects attenuated with age (slopes = -0.0005 to -0.0002), eventually returning to average levels. Anxiety showed a different pattern; nighttime light exposure was unassociated with its intercept and negatively associated with its slope, predicting faster declines in anxiety over time (slope = -0.0003). When reexamining these results at a threshold of p < .001, only the significant effects of nighttime light exposure on baseline ADHD and conduct problems as well as the growth of ADHD, oppositionality, and conduct problems remain. When controlling for relevant covariates, no results remained significant.

**Covariates for Sensitivity Analyses**

The following covariates were controlled for in sensitivity analyses: child gender, race, and ethnicity as well as household income and neighborhood disadvantage. Here we describe how these variables were coded for analysis as covariates (more detailed breakdowns of child and family demographic characteristics are reported in Table 1). For child **gender** and **ethnicity**, dummy-coded variables were created based on parent-reported demographic data (gender: 0 = non-female, 1 = female; ethnicity: 0 = non-Hispanic, 1 = Hispanic/Latino). For **race**, two dummy-coded variables were added to examine associations within the three largest racial categories of our sample: white (0 = non-white, 1 = white) and black/African American (0 = non-black, 1 = black). **Household income** was the parent-reported combined family income from all primary caregivers, with response options binned into 10 categories ranging from 1 (less than $5000) to 10 ($200000 and greater; see Table 1). **Neighborhood disadvantage** was measured with the Area Deprivation Index (ADI; Kind et al., 2014). The ADI considers factors such as education, housing quality, employment, and income. In the ABCD study, the ADI was calculated based on a participant’s census tract (Fan et al., 2021). Weighted summary scores by metropolitan area were used for analyses, with higher scores indicating greater neighborhood deprivation (range 1.07 – 125.75). Rates of missing data for our covariates varied, with less than 1% for child gender and race, 1.29% for child ethnicity, 7.41% for ADI, and 8.56% for household income.

**Patterns of Covariate Effects for Primary Models.** We inspected the associations of all covariates with the latent growth terms (intercepts and slopes) of the main LGCM covariate models (total, internalizing, and externalizing problems). On average, female gender was associated with significantly lower levels of total problems at baseline (Intercepts = -3.61 to -3.60, *p* < .001) and a less steep decline in symptoms over time (Slopes = 0.64, *p* < .001). Identifying as black was associated with significantly lower levels of total problems at baseline (Intercepts = -3.49 to -3.42, *p* < .001) and a faster decline in symptoms over time (Slopes = -0.62 to -0.57, *p* < .01). In addition, having a higher parent-reported income was associated with lower levels of total problems at baseline (Intercepts = -1.35 to -1.32, *p* < .001) and a less steep decline in symptoms over time (Slopes = 0.14, *p* < .001). Identifying as Hispanic/Latino was associated with higher levels of total problems at baseline (Intercepts = 1.57 to 1.75, p < .001) and this effect persisted over time (i.e., no significant effect on the slope). Similarly, higher neighborhood deprivation was associated with higher levels of total problems at baseline (Intercepts = 0.04 to 0.06, *p* < .001) and this effect persisted over time (i.e., nonsignificant effect on the slope).

On average, identifying as Black was associated with lower levels of internalizing problems at baseline (Intercepts = -1.65 to -1.62, *p* < .001) and a faster decline in symptoms over time (Slopes = -0.31 to -0.29, *p* < .001). In addition, having higher parental-reported income was associated with lower levels of internalizing problems at baseline (Intercepts = -0.29 to -0.28, *p* < .001) and a slower decline in symptoms over time (Slopes = 0.03, *p* < .01). Having higher neighborhood deprivation was associated with higher levels of internalizing problems at baseline (Intercepts = 0.01, *p* < .001) and these effects persisted over time (i.e., nonsignificant effect on slope). Interestingly, female gender and identifying as Hispanic/Latino did not have a significant effect on the intercept of internalizing problems but both were associated with a slower decline in symptoms over time (Gender Slopes = 0.33, *p* < .001; Hispanic/Latino Slopes = 0.15 to 0.17, *p* < .01).

On average, female gender was associated with lower levels of externalizing problems at baseline (Intercepts = -1.23 to -1.22, *p* < .001) and a less steep decline in symptoms over time (Slopes = 0.09, *p* < .05). Higher levels of parental-reported income were also associated with lower levels of externalizing problems (Intercepts = -0.47, *p* < .001) and a less steep decline in symptoms over time (Slopes = 0.05, *p* < .001). Identifying as Black was associated with lower levels of externalizing problems at baseline (Intercepts = -0.53 to -0.51, *p* < .05) while identifying as Hispanic/Latino was associated with higher levels (Intercepts = 0.69 to 0.73, *p* < .001). Both of these effects persisted over time (i.e., nonsignificant effects on slopes). Higher neighborhood deprivation was associated with higher levels of externalizing problems at baseline (Intercepts = 0.01, *p* < .001) and this effect also persisted over time.

**References**

Fan, C. C., Marshall, A., Smolker, H., Gonzalez, M. R., Tapert, S. F., Barch, D. M., Sowell, E., Dowling, G. J., Cardenas-Iniguez, C., Ross, J., Thompson, W. K., & Herting, M. M. (2021). Adolescent Brain Cognitive Development (ABCD) study Linked External Data (LED): Protocol and practices for geocoding and assignment of environmental data. *Developmental Cognitive Neuroscience*, *52*, 101030. https://doi.org/10.1016/j.dcn.2021.101030

Kind, A. J. H., Jencks, S., Brock, J., Yu, M., Bartels, C., Ehlenbach, W., Greenberg, C., & Smith, M. (2014). Neighborhood Socioeconomic Disadvantage and 30-Day Rehospitalization. *Annals of Internal Medicine*, *161*(11), 765–774. https://doi.org/10.7326/M13-2946

*
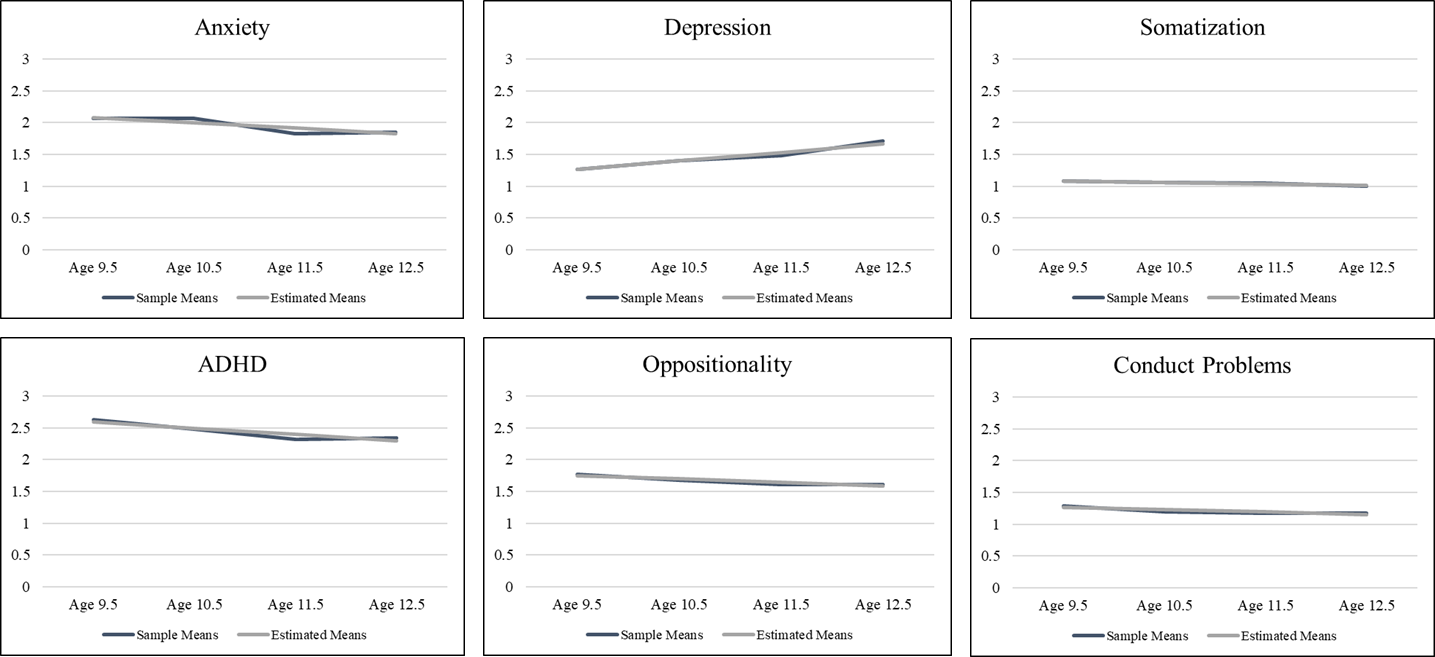
*Figure S1. Observed means and model-implied trajectories for unconditional growth models of symptoms

*Note.* See Table S3 for parameter estimates.

Table S1. Descriptive Statistics of Study Variables

|  | **Baseline Statistics** | | | **Alpha Values** | | | |
| --- | --- | --- | --- | --- | --- | --- | --- |
| **Variables** | **Scale**  **Range** | ***M*** | ***SD*** | ***Baseline*** | ***Year 1*** | ***Year 2*** | ***Year 3*** |
| Total Problems | 0-139 | 18.19 | 17.97 | .95 | .95 | .95 | .95 |
| Internalizing Problems | 0-51 | 5.05 | 5.53 | .87 | .87 | .88 | .89 |
| Externalizing Problems | 0-49 | 4.46 | 5.87 | .90 | .90 | .90 | .90 |
| Anxiety | 0-18 | 2.06 | 2.43 | .77 | .78 | .77 | .78 |
| Depression | 0-26 | 1.27 | 2.01 | .72 | .74 | .76 | .78 |
| Somatic Problems | 0-14 | 1.08 | 1.51 | .64 | .64 | .63 | .64 |
| Attention-Deficit/Hyperactivity Disorder | 0-14 | 2.63 | 2.97 | .85 | .85 | .84 | .84 |
| Oppositionality | 0-10 | 1.77 | 2.04 | .80 | .80 | .80 | .79 |
| Conduct Problems | 0-34 | 1.28 | 2.36 | .82 | .81 | .82 | .81 |
| Green Space: NDVI | 0-1 | 0.44 | 0.36 | - | - | - | - |
| Blue Space: Permanent Water | 0-1 | 0.01 | 0.04 | - | - | - | - |
| Blue Space: Seasonal Water | 0-1 | 0.01 | 0.02 | - | - | - | - |
| Grey Space: Built-Up Land Use | 0-1 | 0.63 | 0.34 | - | - | - | - |
| Grey Space: Nighttime Light | 1-1005 | 98.77 | 84.48 | - | - | - | - |
| Area Deprivation Index | 1-126 | 94.64 | 21.16 | - | - | - | - |

*Note.* NDVI = Normalized Difference Vegetation Index. Scale Range, *M*, and *SD* were all measured at baseline.

^*^ = *p* < .05, ^**^ = *p* < .01, ^***^ = *p* < .001

Table S2. Correlations among Study Variables at Baseline

| **Variables** | **1** | **2** | **3** | **4** | **5** | **6** | **7** | **8** | **9** | **10** | **11** | **12** | **13** | **14** | **15** | **16** | **17** | **18** | **19** |
| --- | --- | --- | --- | --- | --- | --- | --- | --- | --- | --- | --- | --- | --- | --- | --- | --- | --- | --- | --- |
| 1. Total Problems | - |  |  |  |  |  |  |  |  |  |  |  |  |  |  |  |  |  |  |
| 2. Internalizing | .83^***^ | - |  |  |  |  |  |  |  |  |  |  |  |  |  |  |  |  |  |
| 3. Externalizing | .88^***^ | .58^***^ | - |  |  |  |  |  |  |  |  |  |  |  |  |  |  |  |  |
| 4. Anxiety | .74^***^ | .87^***^ | .50^***^ | - |  |  |  |  |  |  |  |  |  |  |  |  |  |  |  |
| 5. Depression | .78^***^ | .79^***^ | .60^***^ | .63^***^ | - |  |  |  |  |  |  |  |  |  |  |  |  |  |  |
| 6. Somatic Problems | .48^***^ | .64^***^ | .31^***^ | .40^***^ | .40^***^ | - |  |  |  |  |  |  |  |  |  |  |  |  |  |
| 7. ADHD | .80^***^ | .49^***^ | .68^***^ | .47^***^ | .53^***^ | .27^***^ | - |  |  |  |  |  |  |  |  |  |  |  |  |
| 8. Oppositionality | .77^***^ | .52^***^ | .89^***^ | .45^***^ | .53^***^ | .28^***^ | .61^***^ | - |  |  |  |  |  |  |  |  |  |  |  |
| 9. Conduct Problems | .73^***^ | .42^***^ | .90^***^ | .35^***^ | .46^***^ | .22^***^ | .56^***^ | .69^***^ | - |  |  |  |  |  |  |  |  |  |  |
| 10. NDVI | -.03^***^ | -.03^***^ | -.03^**^ | -.02^*^ | -.03^***^ | -.02^*^ | -.01 | -.02^*^ | -.02^*^ | - |  |  |  |  |  |  |  |  |  |
| 11. Permanent Water | .01 | .01 | .01 | -.01 | .01 | .02 | .01 | .01 | -.01 | -.03^**^ | - |  |  |  |  |  |  |  |  |
| 12. Seasonal Water | -.01 | -.01 | -.01 | -.01 | -.01 | .01 | .00 | .01 | -.01 | -.01 | .27^***^ | - |  |  |  |  |  |  |  |
| 13. Built-Up Land Use | .03^***^ | .03^**^ | .03^***^ | .02 | .02^*^ | .01 | .02 | .02 | .03^**^ | -.66^***^ | -.15^***^ | -.14^***^ | - |  |  |  |  |  |  |
| 14. Nighttime Light | .05^***^ | .02^*^ | .05^***^ | .02 | .03^**^ | .02 | .05^***^ | .03^**^ | .05^***^ | -.55^***^ | -.06^***^ | -.05^***^ | .59^***^ | - |  |  |  |  |  |
| 15. ADI | .10^***^ | .06^***^ | .10^***^ | .06^***^ | .05^***^ | .05^***^ | .09^***^ | .06^***^ | .11^***^ | .06^***^ | .01 | .01 | -.04^***^ | .13^***^ | - |  |  |  |  |
| 16. Child Gender | -.11^***^ | -.01 | -.12^***^ | -.01 | -.06^***^ | .04^***^ | -.16^***^ | -.11^***^ | -.13^***^ | .01 | -.01 | .01 | .01 | -.01 | .01 | - |  |  |  |
| 17. Child Race (White) | -.03^***^ | .02 | -.05^***^ | .03^**^ | .01 | .01 | -.06^***^ | .01 | -.09^***^ | .13^***^ | .03^**^ | .04^***^ | -.17^***^ | -.21^***^ | -.15^***^ | -.03^**^ | - |  |  |
| 18. Child Race (Black) | .02^*^ | -.05^***^ | .06^***^ | -.05^***^ | -.02^*^ | -.02^*^ | .05^***^ | -.01 | .11^***^ | -.01 | -.03^**^ | -.03^**^ | .10^***^ | .21^***^ | .29^***^ | .02^*^ | -.59^***^ | - |  |
| 19. Child Ethnicity | -.01 | -.03^***^ | .01 | -.04^***^ | -.02^*^ | .00 | -.01 | -.01 | .03^**^ | .32^***^ | .02^*^ | -.03^**^ | -.24^***^ | -.23^***^ | -.05^***^ | -.01 | .06^***^ | .17^***^ | - |
| 20. Household Income | -.17^***^ | -.10^***^ | -.18^***^ | -.08^***^ | -.11^***^ | -.07^***^ | -.13^***^ | -.11^***^ | -.20^***^ | .21^***^ | .04^***^ | .03^**^ | -.21^***^ | -.31^***^ | -.42^***^ | -.01 | .35^***^ | -.39^***^ | .22^***^ |

*Note.* ADHD = Attention-Deficit/Hyperactivity Disorder symptoms, NDVI = Normalized Difference Vegetation Index, ADI = Area Deprivation Index. Please see the *Covariates for Sensitivity Analyses* section for details on how child gender, race, and ethnicity variables were coded for analyses.

^*^ = *p* < .05, ^**^ = *p* < .01, ^***^ = *p* < .001

Table S3. Unconditional latent growth curve model results

| **Symptom Trajectory** | **Estimate** | **S.E.** | **Est./S.E.** | ***p*-value** | **Χ^2^** | **CFI** | **RMSEA** |
| --- | --- | --- | --- | --- | --- | --- | --- |
| **Anxiety** |  |  |  |  | 101.61^***^ | .99 | .04 |
| Means |  |  |  |  |  |  |  |
| Intercept | 2.08 | 0.02 | 95.83 | < .001 |  |  |  |
| Slope | -0.08 | 0.01 | -10.70 | < .001 |  |  |  |
| Variances |  |  |  |  |  |  |  |
| Intercept | 4.23 | 0.08 | 54.53 | < .001 |  |  |  |
| Slope | 0.18 | 0.01 | 15.46 | < .001 |  |  |  |
| Slope-Intercept Correlation | -0.35 | 0.02 | -17.56 | < .001 |  |  |  |
| **Depression** |  |  |  |  | 54.63^***^ | .99 | .03 |
| Means |  |  |  |  |  |  |  |
| Intercept | 1.26 | 0.02 | 69.75 | < .001 |  |  |  |
| Slope | 0.14 | 0.01 | 16.97 | < .001 |  |  |  |
| Variances |  |  |  |  |  |  |  |
| Intercept | 2.94 | 0.06 | 51.83 | < .001 |  |  |  |
| Slope | 0.23 | 0.01 | 19.09 | < .001 |  |  |  |
| Slope-Intercept Correlation | -0.18 | 0.02 | -7.76 | < .001 |  |  |  |
| **Somatization** |  |  |  |  | 38.82^***^ | .99 | .02 |
| Means |  |  |  |  |  |  |  |
| Intercept | 1.08 | 0.01 | 82.71 | < .001 |  |  |  |
| Slope | -0.02 | 0.01 | -4.52 | < .001 |  |  |  |
| Variances |  |  |  |  |  |  |  |
| Intercept | 1.28 | 0.03 | 42.32 | < .001 |  |  |  |
| Slope | 0.05 | 0.01 | 8.85 | < .001 |  |  |  |
| Slope-Intercept Correlation | -0.38 | 0.03 | -14.33 | < .001 |  |  |  |
| **ADHD** |  |  |  |  | 79.43^***^ | .99 | .04 |
| Means |  |  |  |  |  |  |  |
| Intercept | 2.60 | 0.03 | 97.58 | < .001 |  |  |  |
| Slope | -0.10 | 0.01 | -12.75 | < .001 |  |  |  |
| Variances |  |  |  |  |  |  |  |
| Intercept | 6.97 | 0.11 | 61.88 | < .001 |  |  |  |
| Slope | 0.18 | 0.01 | 14.88 | < .001 |  |  |  |
| Slope-Intercept Correlation | -0.38 | 0.02 | -21.04 | < .001 |  |  |  |
| **Oppositionality** |  |  |  |  | 32.98^***^ | .99 | .02 |
| Means |  |  |  |  |  |  |  |
| Intercept | 1.75 | 0.02 | 96.27 | < .001 |  |  |  |
| Slope | -0.06 | 0.01 | -9.24 | < .001 |  |  |  |
| Variances |  |  |  |  |  |  |  |
| Intercept | 3.11 | 0.05 | 58.05 | < .001 |  |  |  |
| Slope | 0.10 | 0.01 | 14.20 | < .001 |  |  |  |
| Slope-Intercept Correlation | -0.38 | 0.02 | -20.35 | < .001 |  |  |  |
| ***Conduct*** |  |  |  |  | 50.51^***^ | .99 | .03 |
| Means |  |  |  |  |  |  |  |
| Intercept | 1.27 | 0.02 | 60.36 | < .001 |  |  |  |
| Slope | -0.04 | 0.01 | -5.14 | < .001 |  |  |  |
| Variances |  |  |  |  |  |  |  |
| Intercept | 4.10 | 0.07 | 57.20 | < .001 |  |  |  |
| Slope | 0.15 | 0.01 | 14.88 | < .001 |  |  |  |
| Slope-Intercept Correlation | -0.38 | 0.02 | -20.26 | < .001 |  |  |  |

*Note.* All estimates are unstandardized except for the slope-intercept correlations, see Figure S1 for plots of observed means and model-implied trajectories.

Table S4. Effects of green space (NDVI) on LGCM symptom trajectory growth terms

| **Effects on Symptom**  **Intercepts and Slopes** | **Estimate** | **S.E.** | **Est./S.E.** | **Χ^2^** | **CFI** | **RMSEA** |
| --- | --- | --- | --- | --- | --- | --- |
| **Anxiety** |  |  |  | 96.96^***^ | .99 | .03 |
| Intercept | -0.15^*^ | 0.06 | -2.39 |  |  |  |
| Slope | 0.06^**^ | 0.02 | 2.82 |  |  |  |
| **Depression** |  |  |  | 56.66^***^ | .99 | .03 |
| Intercept | -0.19^***^ | 0.05 | -3.72 |  |  |  |
| Slope | 0.04 | 0.02 | 1.65 |  |  |  |
| **Somatization** |  |  |  | 39.49^***^ | .99 | .02 |
| Intercept | -0.10^**^ | 0.04 | -2.72 |  |  |  |
| Slope | 0.03^*^ | 0.02 | 2.05 |  |  |  |
| **ADHD** |  |  |  | 71.99^***^ | .99 | .03 |
| Intercept | -0.11 | 0.08 | -1.44 |  |  |  |
| Slope | 0.06^**^ | 0.02 | 2.79 |  |  |  |
| **Oppositionality** |  |  |  | 31.39^***^ | .99 | .02 |
| Intercept | -0.12^*^ | 0.05 | -2.29 |  |  |  |
| Slope | 0.06^***^ | 0.02 | 3.24 |  |  |  |
| **Conduct** |  |  |  | 37.36^***^ | .99 | .02 |
| Intercept | -0.14^*^ | 0.06 | -2.42 |  |  |  |
| Slope | 0.03 | 0.02 | 1.54 |  |  |  |

^*^ = *p* < .05, ^**^ = *p* < .01, ^***^ = *p* < .001

Table S5. Effects of blue space variables on LGCM symptom trajectory growth terms

| **Effects on Symptom**  **Intercepts and Slopes** | **Estimate** | **S.E.** | **Est./S.E.** | **Χ^2^** | **CFI** | **RMSEA** |
| --- | --- | --- | --- | --- | --- | --- |
| **Anxiety** |  |  |  |  |  |  |
| *Permanent Water* |  |  |  | 97.11^***^ | .99 | .03 |
| Intercept | -0.27 | 0.56 | -0.47 |  |  |  |
| Slope | 0.10 | 0.20 | 0.51 |  |  |  |
| *Seasonal Water* |  |  |  | 97.06^***^ | .99 | .03 |
| Intercept | -0.43 | 1.08 | -0.40 |  |  |  |
| Slope | 0.13 | 0.39 | 0.33 |  |  |  |
| **Depression** |  |  |  |  |  |  |
| *Permanent Water* |  |  |  | 54.89^***^ | .99 | .03 |
| Intercept | 0.10 | 0.47 | 0.22 |  |  |  |
| Slope | -0.18 | 0.20 | -0.89 |  |  |  |
| *Seasonal Water* |  |  |  | 55.15^***^ | .99 | .03 |
| Intercept | -0.48 | 0.90 | -0.54 |  |  |  |
| Slope | 0.06 | 0.40 | 0.16 |  |  |  |
| **Somatization** |  |  |  |  |  |  |
| *Permanent Water* |  |  |  | 41.55^***^ | .99 | .02 |
| Intercept | 0.65 | 0.34 | 1.91 |  |  |  |
| Slope | -0.17 | 0.13 | -1.26 |  |  |  |
| *Seasonal Water* |  |  |  | 41.13^***^ | .99 | .02 |
| Intercept | 0.74 | 0.65 | 1.13 |  |  |  |
| Slope | -0.15 | 0.27 | -0.57 |  |  |  |
| **ADHD** |  |  |  |  |  |  |
| *Permanent Water* |  |  |  | 73.43^***^ | .99 | .03 |
| Intercept | 0.02 | 0.70 | 0.03 |  |  |  |
| Slope | 0.12 | 0.20 | 0.58 |  |  |  |
| *Seasonal Water* |  |  |  | 73.03^***^ | .99 | .03 |
| Intercept | 0.29 | 1.33 | 0.22 |  |  |  |
| Slope | 0.60 | 0.40 | 1.52 |  |  |  |
| **Oppositionality** |  |  |  |  |  |  |
| *Permanent Water* |  |  |  | 31.28^***^ | .99 | .02 |
| Intercept | 0.44 | 0.47 | 0.92 |  |  |  |
| Slope | -0.28 | 0.15 | -1.83 |  |  |  |
| *Seasonal Water* |  |  |  | 31.25^***^ | .99 | .02 |
| Intercept | 0.37 | 0.90 | 0.41 |  |  |  |
| Slope | -0.21 | 0.30 | -0.69 |  |  |  |
| **Conduct** |  |  |  |  |  |  |
| *Permanent Water* |  |  |  | 37.95^***^ | .99 | .02 |
| Intercept | -0.07 | 0.54 | -0.14 |  |  |  |
| Slope | 0.06 | 0.18 | 0.31 |  |  |  |
| *Seasonal Water* |  |  |  | 37.56^***^ | .99 | .02 |
| Intercept | -0.85 | 1.03 | -0.83 |  |  |  |
| Slope | 0.31 | 0.35 | 0.88 |  |  |  |

^*^ = *p* < .05, ^**^ = *p* < .01, ^***^ = *p* < .001

Table S6. Effects of grey space variables on LGCM symptom trajectory growth terms

| **Effects on Symptom**  **Intercepts and Slopes** | **Estimate** | **S.E.** | **Est./S.E.** | **Χ^2^** | **CFI** | **RMSEA** |
| --- | --- | --- | --- | --- | --- | --- |
| **Anxiety** |  |  |  |  |  |  |
| *Built-Up Land Use* |  |  |  | 99.13^***^ | .99 | .03 |
| Intercept | 0.12 | 0.07 | 1.84 |  |  |  |
| Slope | -0.03 | 0.02 | -1.20 |  |  |  |
| *Nighttime Lights* |  |  |  | 103.03^***^ | .99 | .04 |
| Intercept | 0.0003 | 0.000 | 1.04 |  |  |  |
| Slope | -0.0003^*^ | 0.000 | -2.54 |  |  |  |
| **Depression** |  |  |  |  |  |  |
| *Built-Up Land Use* |  |  |  | 62.06^***^ | .99 | .03 |
| Intercept | 0.14^**^ | 0.06 | 2.59 |  |  |  |
| Slope | -0.02 | 0.02 | -0.86 |  |  |  |
| *Nighttime Lights* |  |  |  | 56.59^***^ | .99 | .03 |
| Intercept | 0.001^**^ | 0.000 | 3.10 |  |  |  |
| Slope | -0.0003^**^ | 0.000 | -2.86 |  |  |  |
| **Somatization** |  |  |  |  |  |  |
| *Built-Up Land Use* |  |  |  | 39.68^***^ | .99 | .02 |
| Intercept | 0.04 | 0.04 | 1.01 |  |  |  |
| Slope | -0.01 | 0.02 | -0.57 |  |  |  |
| *Nighttime Lights* |  |  |  | 40.38^***^ | .99 | .02 |
| Intercept | 0.0004^*^ | 0.000 | 2.31 |  |  |  |
| Slope | -0.0002^**^ | 0.000 | -2.67 |  |  |  |
| **ADHD** |  |  |  |  |  |  |
| *Built-Up Land Use* |  |  |  | 74.74^***^ | .99 | .03 |
| Intercept | 0.14 | 0.08 | 1.73 |  |  |  |
| Slope | -0.06^**^ | 0.02 | -2.63 |  |  |  |
| *Nighttime Lights* |  |  |  | 76.67^***^ | .99 | .03 |
| Intercept | 0.002^***^ | 0.000 | 4.73 |  |  |  |
| Slope | -0.0005^***^ | 0.000 | -4.95 |  |  |  |
| **Oppositionality** |  |  |  |  |  |  |
| *Built-Up Land Use* |  |  |  | 33.09^***^ | .99 | .02 |
| Intercept | 0.09 | 0.06 | 1.56 |  |  |  |
| Slope | -0.03 | 0.02 | -1.69 |  |  |  |
| *Nighttime Lights* |  |  |  | 31.62^***^ | .99 | .02 |
| Intercept | 0.001^**^ | 0.000 | 2.85 |  |  |  |
| Slope | -0.0003^***^ | 0.000 | -3.48 |  |  |  |
| **Conduct** |  |  |  |  |  |  |
| *Built-Up Land Use* |  |  |  | 38.04^***^ | .99 | .02 |
| Intercept | 0.18^**^ | 0.06 | 2.80 |  |  |  |
| Slope | -0.03 | 0.02 | -1.44 |  |  |  |
| *Nighttime Lights* |  |  |  | 37.47^***^ | .99 | .02 |
| Intercept | 0.001^***^ | 0.000 | 5.48 |  |  |  |
| Slope | -0.0003^***^ | 0.000 | -3.42 |  |  |  |

^*^ = *p* < .05, ^**^ = *p* < .01, ^***^ = *p* < .001
